# EFFECTS OF EPISTASIS ON INFECTIVITY RANGE DURING HOST-PARASITE COEVOLUTION

**DOI:** 10.1111/evo.12479

**Published:** 2014-07-25

**Authors:** Ben Ashby, Sunetra Gupta, Angus Buckling

**Affiliations:** 1 Department of Zoology, University of Oxford, South Parks RoadOxford, OX1 3PS, United Kingdom; 3 Biosciences, University of Exeter, Penryn CampusTR10 9FE, United Kingdom

**Keywords:** Epistasis, generalism, host-parasite coevolution, infectivity range, multiple mutations, resistance

## Abstract

Understanding how parasites adapt to changes in host resistance is crucial to evolutionary epidemiology. Experimental studies have demonstrated that parasites are more capable of adapting to gradual, rather than sudden changes in host phenotype, as the latter may require multiple mutations that are unlikely to arise simultaneously. A key, but as yet unexplored factor is precisely how interactions between mutations (epistasis) affect parasite evolution. Here, we investigate this phenomenon in the context of infectivity range, where parasites may experience selection to infect broader sets of genotypes. When epistasis is strongly positive, we find that parasites are unlikely to evolve broader infectivity ranges if hosts exhibit sudden, rather than gradual changes in phenotype, in close agreement with empirical observations. This is due to a low probability of fixing multiple mutations that individually confer no immediate advantage. When epistasis is weaker, parasites are more likely to evolve broader infectivity ranges if hosts make sudden changes in phenotype, which can be explained by a balance between mutation supply and selection. Thus, we demonstrate that both the rate of phenotypic change in hosts and the form of epistasis between mutations in parasites are crucial in shaping the evolution of infectivity range.

Antagonistic coevolution between hosts and parasites can lead to directional selection for more effective defense and counter-defense mechanisms (Thrall and Burdon 2003; Labrie et al. 2010; Schulte et al. 2010; Brown and Tellier 2011). In many cases, these dynamics (often referred to as “coevolutionary arms races”) are characterized by reciprocal expansions in the range of genotypes that the host can resist and the parasite can infect, which means that populations tend to fare better than their ancestors when confronted with contemporary antagonists (Buckling and Rainey 2002; Mizoguchi et al. 2003; Thrall and Burdon 2003; Brown and Tellier 2011; Scanlan et al. 2011). Understanding precisely why some parasites develop broader infectivity ranges than others has important implications for our ability to predict how parasites will evolve in response to shifting patterns of host resistance or other environmental changes, with particular relevance for the use of biocontrol in industry and medicine (Tait et al. 2002; Levin and Bull 2004). While variation in infectivity range is typically explained by selection (e.g., fitness costs; Fenton and Brockhurst 2007; Ashby et al. 2014) or fundamental genetic constraints (e.g., parasites may be forced to specialize on one group of hosts or another; Dybdahl and Lively 1998; Decaestecker et al. 2007; Koskella and Lively 2007), a lack of broad infectivity ranges may also result from the need to fix multiple, rather than single, mutations (Benmayor et al. 2009; Paterson et al. 2010; Hall et al. 2011; Meyer et al. 2012). Here, we investigate how key parameters (epistasis and the rate of phenotypic change in the host) affect the fixation of multiple mutations, and hence infectivity range, during coevolution.

Parasites frequently require multiple amino acid substitutions to infect a novel host, and the likelihood of several beneficial mutations occurring simultaneously or in quick succession is usually slim (Benmayor et al. 2009; Hall et al. 2011; Scanlan et al. 2011; Gururani et al. 2012; Meyer et al. 2012; Russell et al. 2012). In some cases, however, subsets of mutations may confer an immediate fitness advantage on contemporaneous hosts, increasing the probability that a complete set will eventually become fixed (Meyer et al. 2012). Empirical observations using bacteria and viruses suggest that these conditions are most likely to be realized when parasites are exposed to genetically diverse host populations, such that they experience gradual, rather than sudden changes in phenotype during coevolution, as individual mutations may increase performance on subsets of the host population (Hall et al. 2011; Meyer et al. 2012). Crucially, a wide range of genetic and ecological processes, such as recombination and gene flow, could alter the rate of phenotypic change in the host population (Sasaki 2000; Gandon 2002; Gandon and Nuismer 2009), and hence the likelihood of broad infectivity ranges evolving.

Empirical studies that demonstrate the importance of coevolution for the emergence of broad infectivity ranges have used host-parasite interactions that are governed by strong positive epistasis between infectivity mutations (Paterson et al. 2010; Hall et al. 2011; Meyer et al. 2012). This means that parasites with an incomplete set of mutations will fare no better (or even worse) on the novel host than parasites with none. However, both quantitatively and qualitatively different forms of epistasis governing infectivity have been identified, including weak positive, negative, and no epistasis (Lenski 1984; Wilfert and Schmid-Hempel 2008), and it is currently unclear how this will impact on infectivity range during coevolution. Here, we demonstrate theoretically that different forms of epistasis have contrasting effects on the ability of parasites to expand their infectivity ranges when hosts exhibit gradual or sudden changes in phenotype during coevolution. Our results are in good agreement with empirical observations when epistasis is strongly positive (gradual changes in host phenotype promote broader infectivity ranges), but notably, we find that the opposite outcome is expected for weaker forms of epistasis (sudden changes in host phenotype promote broader infectivity ranges).

## Methods

### MODEL DESCRIPTION

**Figure 1:**
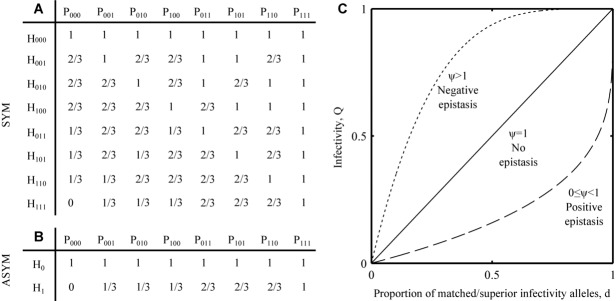
Genetic interactions between hosts and parasites. The tables show the proportion of parasite loci, *d*, that match or are superior to (a) corresponding loci (symmetric [SYM] scenario) or (b) the sole locus (asymmetric [ASYM] scenario) in the host. Interactions between hosts (H) and parasites (P) are shown for *n* = 3, where subscripts correspond to the presence (1) or absence (0) of a resistance or infectivity allele at a given locus. (c) Infectivity (Q; eq. 1) as a function of *d* for different values of the epistasis parameter, ψ, which modifies the type and strength of epistasis between infectivity alleles: 0 ≤ ψ < 1, ψ > 1 and ψ = 1 give positive, negative, and no epistasis, respectively.

We compare two types of genetic specificity that govern host-parasite interactions, both of which allow the evolution of parasites with broader infectivity ranges. The first is similar to the multilocus gene-for-gene framework proposed by Sasaki (2000), with interactions occurring at *n* biallelic loci in both host and parasite. Increasing the number of infectivity alleles improves infectivity to a wider range of host genotypes, and increasing the number of resistance alleles improves resistance to a wider range of parasite genotypes. We refer to this as a “symmetric” (SYM) interaction, because there is a one-to-one correspondence between resistance and infectivity alleles. We compare this scenario to an “asymmetric” (ASYM) form of genetic specificity, where interactions occur between a single locus in the host and *n* loci in the parasite (one-to-many). In this case, there are only two possible host genotypes (susceptible and resistant), and increasing the number of infectivity alleles can improve performance on the resistant host. Genotypes are represented by binary strings (hosts: 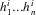 (SYM) or 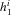 (ASYM); parasites: 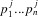; superscripts identify each genotype), where each locus corresponds to the presence (1) or absence (0) of a resistance (host) or infectivity (parasite) allele. Infectivity alleles interact with each other and with resistance alleles to modulate the overall strength of infectivity, *Q*, on a given host, such that: 
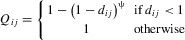
1where 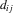 is the proportion of infectivity alleles that match or exceed either (i) the resistance allele at each corresponding locus (SYM: 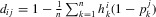), or (ii) the sole resistance allele in the host (ASYM: 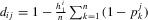) (Fig.1A–B). The parameter ψ modulates the type and strength of epistasis between infectivity alleles, such that 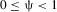, 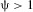 and 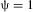 give positive, negative and no epistasis, respectively (Fig.1C). Values of ψ further away from 1 give stronger forms of epistasis; in the special case of 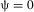 infection is only possible if 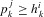 at all loci.

We base the epidemiological dynamics of our model on the SI framework, where hosts of genotype *i* are classed as either susceptible 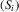 or infected by parasite genotype *j* 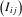. Hosts are haploid and reproduce asexually with a maximum per-capita birth rate of 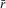, and experience a density-dependent per-capita mortality rate of at least 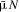, where 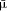 measures the strength of competition for resources and *N* is the total population size. We set 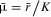, so that the host population tends towards a carrying capacity of *K* in the absence of disease. Initial populations are composed of *K* susceptible and 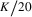 infected hosts, with no resistance or infectivity alleles present. The host population mixes randomly and exhibits either frequency- (FD) or density-dependent (DD) contact patterns, so that a susceptible host of genotype *i* will be infected with parasite *j* at a rate of 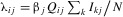 (FD) or 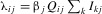 (DD) per unit time, where 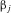 is the transmission coefficient of the parasite (base transmission coefficient: 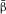). Infected hosts are unable to recover and suffer an increased mortality rate, given by the parameter 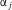 (base disease-associated mortality rate: 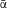) coinfection does not occur. New generations are subject to mutation rates of 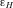 and 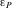 at each locus for hosts and parasites, respectively, with the restriction that multiple mutations cannot arise simultaneously (i.e., the genotypes of parent and progeny never differ at more than one locus).

Broader resistance and infectivity ranges are often associated with a fitness cost (Chao et al. 1977; Webster and Woolhouse 1999; Bohannan et al. 2002; Poullain et al. 2008), which we incorporate into either the host per-capita birth rate 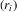 or the coefficient of density-dependent mortality 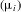, and either the disease-associated mortality rate 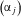 or transmission coefficient 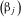 for parasites. We limit simulations to one type of fitness cost per population, giving a total of four combinations. For example, if hosts experience a fitness cost in the form of a reduced birth rate, then the coefficient of density-dependent mortality remains constant for all genotypes 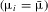 and if parasites with broader infectivity ranges have a lower transmission coefficient, then the disease-associated mortality rate does not vary 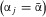. When fitness costs do affect a particular life-history trait, they do so based on the following equations: 
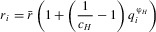
2a

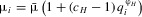
2b

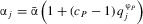
2c

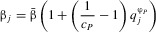
2dwhere for hosts (parasites), 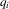 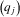 gives the proportion of loci that contain a resistance (infectivity) allele, 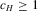 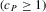 is the maximum strength of the fitness cost and 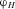 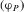 controls whether costs are accelerating 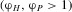, decelerating 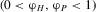 or linear 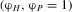. Note that for 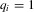, 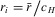 or 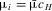, which means that the birth rate or coefficient of density-dependent mortality is 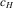 times lower/higher for individuals with a full complement of resistance alleles than the base values (similarly for 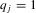: 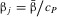 and 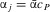). Although resistance and infectivity alleles can behave epistatically for both specificity (*Q*) and fitness costs (see e.g., Fenton and Brockhurst 2007), we shall only refer to epistasis in the context of the parasite's ability to infect a given host (i.e., in terms of the parameter ψ) to avoid confusion. Variations in ψ will be referred to as positive, negative or no epistasis, whereas variations in 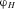 and 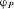 will be referred to accelerating, decelerating or linear fitness costs.

The dynamics of our model (excluding mutations) are captured by the following set of coupled ordinary differential equations: 
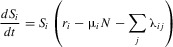
3a

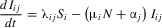
3b

We translate this deterministic framework into a stochastic model by using the τ-leap method proposed by Gillespie (2001), which uses a fixed step size, τ, and assumes the number of events occurring within a time step is Poisson distributed. The optimal genotype will always emerge in a deterministic framework with no extinction threshold, but we should expect parasites to struggle to accumulate infectivity alleles when demographic stochasticity is included, especially if resistance spreads rapidly. Thus, by comparing the deterministic and stochastic models, we are able to establish if broader infectivity ranges do not evolve due to selection (i.e., broader infectivity ranges are not beneficial), or if mutations are struggling to reach fixation due to stochasticity.

### ANALYSIS

We analyze the deterministic and stochastic versions of our model to evaluate how the previously described forms of genetic specificity (SYM and ASYM) and different types of epistasis (ψ) influence the evolution of broader infectivity ranges. In other words, we establish how these genetic factors affect the ability of parasites to accumulate infectivity alleles. At each time point, we measure the average proportion of parasite loci that contain an infectivity allele and define “peak infectivity range,” *E*, to be the maximum of this value over the course of a simulation (20,000 time units). Thus, if 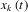 is the proportion of parasites that have a total of *k* infectivity alleles at time *t*, then: 
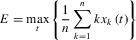
8

We measure the maximum value over the duration of each simulation as GFG frameworks can produce fluctuations in range (e.g., Sasaki 2000), but the focus of the present study is whether genetic factors affect the initial emergence of broader infectivity ranges and not whether they are evolutionarily stable. We wish to determine the general behavior of our model, but since we are not modeling a particular host-parasite system, the parameter space is somewhat arbitrary. To overcome this issue, we fix τ = 0.1 and vary ψ, 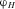 and 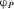 incrementally to cover all qualitatively different forms of epistasis (positive, negative, or none) and fitness costs (accelerating, decelerating, or linear), and use a Latin hypercube sample (LHS) to draw the remaining parameters from the distributions in Table1, the majority of which are varied over at least an order of magnitude, covering biologically plausible areas of parameter space (e.g., population sizes of 10^5^–10^9^ are appropriate for microbial communities). Note that the mutation rates and the coefficient of density-dependent mortality are not used in the construction of the LHS, but are instead fixed by the base per-capita birth rate and/or carrying capacity, which are part of the LHS design. The parameters 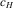 and 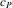 vary over relatively narrow ranges compared to the other parameters as previous studies have demonstrated that high fitness costs greatly limit infectivity range in well-mixed populations (Sasaki 2000; Ashby et al. 2014). The LHS contains 1000 parameter combinations, each of which is tested in both SYM and ASYM scenarios with all four possible combinations of fitness costs (eq. 2) for different values of ψ, 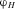, and 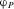. This method allows us to determine if one form of genetic specificity (SYM or ASYM) consistently allows broader infectivity ranges to evolve than the other, and if this relationship holds for all forms of epistasis (ψ) and fitness costs (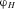 and 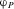). We discard simulations where the parasite dies out in either the SYM or ASYM scenarios for a given set of parameters.

**Table 1:** Parameter distributions used for the Latin hypercube sample

	Description	Range
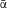	Base disease-associated mortality rate	(0.01–0.1)
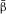	Base transmission coefficient	FD: (0.1–1), DD: (10^—9^–10^–5^)*
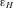	Host mutation rate	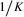 **
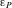	Parasite mutation rate	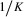 **
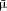	Base coefficient of density-dependent mortality	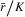 **
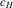	Maximum strength of host fitness costs	(1.05–1.2)
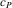	Maximum strength of parasite fitness costs	(1.05–1.5)
*K*	Carrying capacity	(10^5^–10^9^)
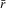	Base per-capita birth rate	(0.01–0.1)

* FD, frequency-dependent transmission; DD, density-dependent transmission.

** Values are fixed by the base per-capita birth rate and/or carrying capacity.

## Results

For the sake of brevity, here we only present results for parasite populations with three loci 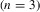, contact patterns based on frequency-dependence (FD) and decelerating fitness costs 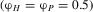 affecting only the per-capita birth rate 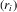 and transmission coefficient 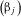. However, the results are qualitatively similar for other numbers of loci (Supplementary Fig. S1), density-dependent contact patterns (Supplementary Fig. S2), linear and accelerating fitness costs (Supplementary Fig. S3) and different combinations of cost functions (Supplementary Fig. S4).

### ALGEBRAIC ANALYSIS

Using equation (3), we can derive the basic reproductive ratio, 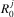, for a parasite in a naive, fully susceptible host population when transmission is frequency-dependent: 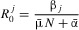
9

In the absence of resistant hosts, this quantity is maximized when the parasite has no infectivity alleles. We can also derive the effective basic reproductive ratio, 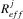, which is the average number of secondary infections produced for any composition of hosts: 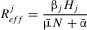
10where 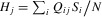 is the proportion of the host population that the parasite is able to infect. For a given composition of hosts, parasite *j* should initially perform better than the wild type (no infectivity alleles, *H*_0_) provided 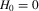 and 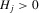, or, for 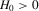: 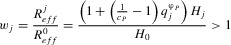
11

**Figure 2:**
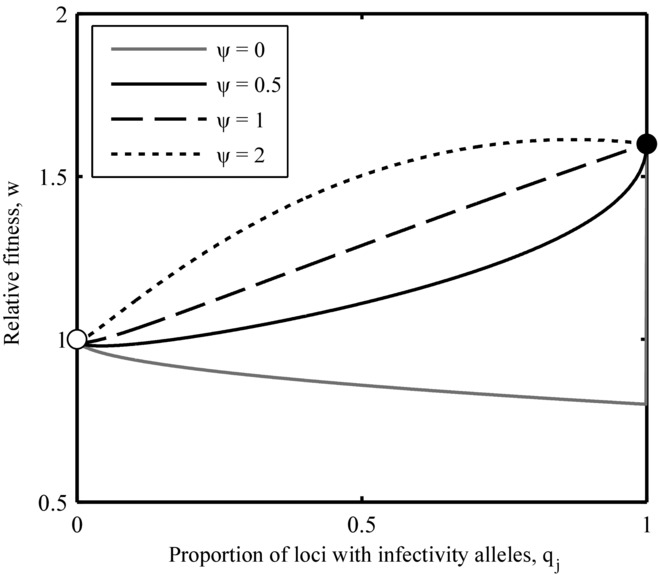
Relative fitness (w; eq. 11) of parasites in a fixed host population for different values of the epistasis parameter, ψ. The white circle indicates the fitness of the wild-type parasite (no infectivity alleles) and the black circle indicates the fitness of parasites with a full complement of infectivity alleles. When epistasis is strongly positive (gray), parasites with a complete set of infectivity alleles may have the highest fitness, but intermediate genotypes could perform worse than the wild type, so parasites may struggle to accumulate infectivity alleles (note the step-change when q_j_ = 1 for ψ = 0). When epistasis is weaker (solid black) or nonexistent (dashed), parasites with an incomplete set of infectivity alleles are likely to experience an immediate increase in fitness, which may allow infectivity ranges to expand. When epistasis is negative (dotted), an incomplete set of mutations may be optimal due to the presence of fitness costs, which outweigh the benefits of broader infectivity ranges. Parameters: φ_P_ = 0.5; c_P_ = 1.25.

Figure2 shows several examples of how this fitness function varies with the number of infectivity alleles in the parasite for different values of the epistasis parameter, ψ. While Figure2 represents an idealized scenario with the host population held constant (50% wild type, 50% maximal resistance), it does reveal some interesting patterns. In particular, parasites with an intermediate number of infectivity alleles may perform worse than the wild type if epistasis is positive. This suggests that broad infectivity ranges are unlikely to emerge if hosts make sudden jumps in phenotype. Clearly, the composition of the host population will alter selection among parasites and vice versa, which prevents further algebraic analysis of this system. In addition, real populations are subject to stochasticity, which can have a considerable influence on the accumulation of rare mutations. However, based on the above analysis, we can make two predictions to test numerically: (i) selection for parasites with broad infectivity ranges should peak for low values of ψ, as complete or near complete sets of mutations are required to overcome host resistance; (ii) parasites in the ASYM scenario will struggle to accumulate mutations when demographic stochasticity is included if epistasis is strongly positive, as intermediate genotypes may perform worse than the wild type.

### NUMERICAL ANALYSIS

#### Strong positive epistasis selects for parasites with broad infectivity ranges

**Figure 3:**
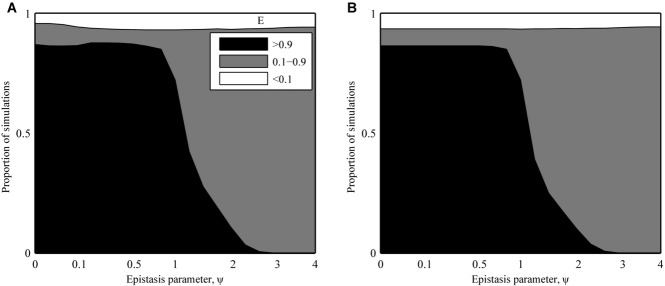
Proportion of deterministic simulations where peak infectivity range (E; eq. 8) was greater than 0.9 (black), less than 0.1 (white), or between these values (gray), for (A) gradual (SYM) and (B) sudden (ASYM) changes in host phenotype. The parameter ψ controls the type and strength of epistasis between infectivity alleles, ranging from strong positive (ψ << 1), through weak positive (ψ < 1), none (ψ = 1), and finally, negative (ψ > 1) epistasis. The fittest genotype always emerges in a deterministic framework with no extinction threshold, so the SYM and ASYM scenarios produce almost identical outputs.

Numerical analysis of the deterministic model revealed that infectivity range peaks when epistasis is positive (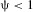; Fig.3), but declines rapidly if epistasis is negative 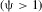. The lack of an extinction threshold in the deterministic model means that the optimal genotype is always able to emerge, so there are no qualitative differences between the SYM and ASYM scenarios.

#### Stochasticity constrains infectivity range when hosts exhibit sudden changes in phenotype

**Figure 4:**
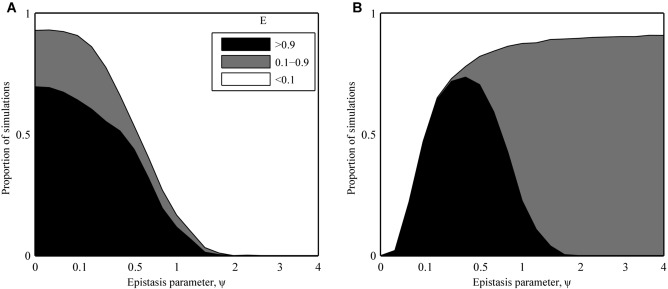
Proportion of stochastic simulations where peak infectivity range (E; eq. 8) was greater than 0.9 (black), less than 0.1 (white), or between these values (gray), for (a) gradual (SYM) and (b) sudden (ASYM) changes in host phenotype. The parameter ψ controls the type and strength of epistasis between infectivity alleles, ranging from strong positive (ψ << 1), through weak positive (ψ < 1), none (ψ = 1), and finally, negative (ψ > 1) epistasis. The patterns in the SYM scenario are broadly similar to those in the deterministic version of the model (Fig.3A), but infectivity range is predicted to peak for weak positive epistasis in the ASYM scenario. This disparity can be explained by the low probability of fixing multiple mutations in the presence of strong positive epistasis.

The pattern of parasite evolution in the stochastic SYM scenario was broadly similar to that described for the deterministic model: infectivity ranges peaked for strong positive epistasis 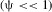 and decreased with greater ψ (Fig.4A). Yet, unlike the deterministic version of the SYM scenario, the stochastic version exhibited very little, if any, selection for broader infectivity ranges for negative epistasis 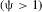. This is due to reduced selection for resistance in the host (Supplementary Fig. S5C). When epistasis between infectivity mutations is negative, resistance is largely ineffective as hosts require multiple mutations to achieve a significant reduction in susceptibility. Hence, hosts do not tend to evolve broader resistance ranges when demographic stochasticity is included and consequently parasites do not need to evolve broader infectivity ranges under these conditions.

In contrast, the stochastic version of the ASYM scenario produced markedly different outcomes to both the deterministic model and the SYM scenario (Fig.4B). Specifically, broad infectivity ranges were extremely rare for strong positive epistasis and instead peaked for weaker interactions (intermediate values of ψ). This pattern was consistent for different types of fitness cost, variations in the rate at which fitness costs increased (i.e., accelerating, decelerating, or linear), different numbers of loci and density-dependent transmission (see Supplementary Material).

We extended the duration of 10% of our stochastic simulations to 200,000 time steps (a tenfold increase) to ensure that the differences between the two versions of the ASYM scenario were not attributable to faster evolution in the deterministic setting. However, longer simulations did not change the overall pattern of our results and only led to relatively minor quantitative differences (average change in *E*: 0.03). Notably, parasites that experienced very strong positive epistasis (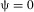) were still unable to accumulate infectivity alleles, even over this longer time period.

**Figure 5:**
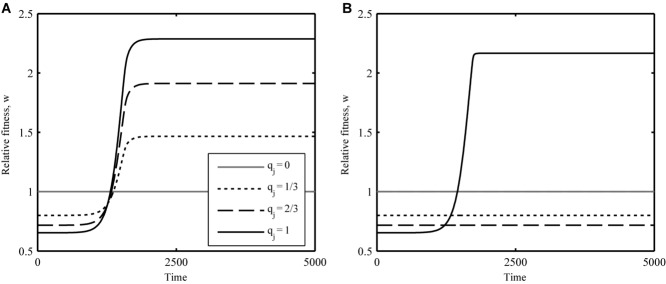
Example dynamics showing the relative fitness (w; eq. 11) of parasites with different proportions of loci containing infectivity alleles (q_j_ = 0 (gray), 1/3 (dotted), 2/3 (dashed), and 1 (solid)) for (A) gradual (SYM) and (B) sudden (ASYM) changes in host phenotype (deterministic version). (A) When resistance begins to spread in a host population that exhibits gradual changes in phenotype (around 1000 time units) parasites with incomplete sets of infectivity alleles experience an immediate increase in fitness, which allows these mutations to be fixed, leading to the evolution of broad infectivity ranges. (B) If the host makes a sudden change in phenotype, then parasites with incomplete sets of infectivity alleles may not experience an immediate increase in fitness. Hence, infectivity alleles may not accumulate when demographic stochasticity is included, even though parasites with a full complement of infectivity alleles have the highest fitness. Parameters: α = 0.01; β = 0.25; ε_H_ = 10^−6^; ε_P_ = 10^−6^; φ_H_ = 0.5; φ_P_ = 0.5; ψ = 0; c_H_ = 1; c_P_ = 1.25; K = 10^6^; *n* = 3; *r* = 0.05.

The disparity between the deterministic and stochastic versions in the ASYM scenario can be explained by the low probability of fixing multiple mutations that were characterized by strong positive epistasis (i.e., parasites were trapped at a local fitness peak). Thus, although a complete set of infectivity alleles may have been optimal for low values of ψ (Figs.2, 3B), parasites struggled to accumulate mutations that were not immediately beneficial. This situation did not occur in the SYM scenario, as individual mutations conferred an immediate increase in fitness due to the presence of genetically intermediate hosts (hosts exhibited gradual changes in phenotype). Figure5 shows contrasting dynamics from the two scenarios. When hosts exhibit gradual changes in phenotype (SYM; Fig.5A), the fitness of parasites with incomplete sets of infectivity alleles is greater than the wild type, which allows individual mutations to become fixed. When hosts exhibit sudden changes in phenotype (ASYM; Fig.5B), parasites with the broadest ranges still have the highest fitness, but an incomplete set of infectivity alleles is costly, so mutations are unlikely to accumulate when demographic stochasticity is included.

Although broad infectivity ranges were much more common in the stochastic version of the SYM scenario than in the corresponding ASYM scenario for strong positive epistasis 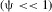, the converse was true for weak positive epistasis 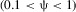. This pattern can again be explained by less effective resistance in the host as epistasis between infectivity alleles weakens (Fig. S5C). Gradual changes in phenotype are less advantageous to the host as ψ increases, reducing the likelihood that resistance alleles will become fixed in a stochastic setting, which in turn reduces selection for parasites with broader infectivity ranges. Thus, for weak positive epistasis, overall levels of resistance will tend to be lower if hosts are restricted to gradual (SYM) rather than sudden (ASYM) changes in phenotype (SYM).

## Discussion

Our study was inspired by recent experiments where gradual changes in host phenotype provided optimal conditions for parasite evolution, presumably by facilitating the accumulation of multiple mutations in quick succession, whereas sudden changes in host phenotype prevented mutations from being fixed (Benmayor et al. 2009; Paterson et al. 2010; Hall et al. 2011; Meyer et al. 2012). However, we hypothesized that the presence of strong positive epistasis between mutations was likely to be a key factor in these experiments and that alternative forms of epistasis could lead to different evolutionary outcomes. Using a theoretical approach, we explored how the rate of phenotypic change in the host (gradual vs. sudden) and the type and strength of epistasis shape the evolution of broader infectivity ranges. Our findings support empirical observations that gradual changes in host phenotype promote broad infectivity ranges, provided epistasis is strongly positive (Paterson et al. 2010; Hall et al. 2011; Meyer et al. 2012). Moreover, by comparing deterministic and stochastic models, we have shown why sudden changes in host phenotype can restrict parasite evolution (low probabilities of fixing individual mutations that confer no immediate increase in fitness). However, we have also shown that this prediction does not hold for weak positive epistasis: parasites are more likely to evolve broad infectivity ranges if hosts exhibit sudden changes in phenotype. These results demonstrate that the nature of epistasis can be crucial for shaping parasite evolution and that gradual changes in host phenotype may not always provide the optimal conditions for broad infectivity ranges to evolve.

When epistasis is strongly positive, parasites require a complete (or near-complete) set of mutations to overcome resistance. These are difficult to accumulate if the host makes sudden changes in phenotype (e.g., due to the loss of a key receptor) as individual mutations may carry an intrinsic fitness cost without conferring any benefits with regards to increased infectivity. If, however, hosts experience gradual changes in phenotype (e.g., reduced expression of a key receptor), then parasites may have access to hosts that are phenotypically intermediate between ancestral and future populations. Individual mutations may then confer an immediate fitness advantage, dramatically increasing the probability that multiple mutations will become fixed. Hence the symmetric (SYM) scenario in our model, which featured intermediate hosts, was much more favorable to the emergence of broad infectivity ranges under strong positive epistasis than the asymmetric (ASYM) scenario, which did not allow intermediate hosts to evolve. Conversely, when epistasis is negative there is a diminishing benefit associated with the acquisition of multiple infectivity alleles regardless of the potential presence of intermediate hosts. In other words, parasites can infect a reasonably broad set of hosts with a single mutation (e.g., Jonah et al. 2003; Brault et al. 2004) and the advantages of a full complement of mutations are outweighed by associated fitness costs. Between these extremes (i.e., for weak positive epistasis), individual infectivity alleles may confer a slight increase in fitness, allowing parasites to accumulate successive mutations. These mutations are likely to be under strong selection when hosts exhibit sudden changes in phenotype, but will be less beneficial if hosts evolve more gradually. Hence, infectivity range peaks for weak positive epistasis in our ASYM scenario and is more common than in the SYM scenario under these conditions.

The results for the ASYM scenario can also be interpreted in terms of a balance between mutation supply and selection for broader infectivity ranges. If parasites perform poorly on current hosts, then selection for broader infectivity ranges is strong, but the mutation supply is constrained due to a lack of suitable hosts. This problem is accentuated when epistasis between infectivity alleles is strongly positive, as multiple mutations that may be costly in isolation are required before parasites can infect large numbers of hosts. Hence, strong positive epistasis results in a low mutation supply and broader infectivity ranges are unlikely to evolve. Conversely, if parasites perform well on the current host population, then the mutation supply is much greater, but selection for broader infectivity ranges is inevitably weaker. When epistasis is negative, parasites with few infectivity alleles can perform reasonably well on hosts that have invested in resistance; accumulating further (costly) infectivity alleles is unlikely to be beneficial under these circumstances, so again, broader infectivity ranges do not evolve. Between these extremes, parasites can infect sufficient hosts to maintain a modest supply of mutations while experiencing fairly strong selection for broader infectivity ranges. Hence, broader infectivity ranges are most likely to evolve in the stochastic ASYM scenario for intermediate values of ψ, which corresponds to weak positive epistasis.

The discrepancies between the deterministic and stochastic versions of the ASYM scenario are striking, but were not attributable to different rates of evolution, as simulations that were allowed to run for much longer time periods produced very similar results (although over infinitely long time scales or with simultaneous mutations, the stochastic model should eventually produce pathogens with complete sets of infectivity alleles when epistasis is strongly positive). If hosts make sudden jumps in phenotype, then parasites that experience stochasticity and strong positive epistasis between infectivity alleles are likely to get stuck at a local fitness peak. In the deterministic model, parasites are able to explore the entire fitness landscape, which always allows the globally optimal phenotype to emerge. The contrasting outcomes in the deterministic and stochastic models highlight the need to compare different modeling approaches when studying host-parasite coevolution, as optimal phenotypes may not always emerge when demographic stochasticity is included (see also Ashby et al. 2014). It is important to note that our approach differs from most other theoretical studies of host-parasite coevolution, which typically omit ecological feedbacks and stochasticity, assume that population sizes are infinite and focus only on changes in gene frequencies (Sasaki 2000; Agrawal and Lively 2002, 2003; Fenton and Brockhurst 2007; Fenton et al. 2009, 2012). As a consequence, these studies do not explicitly model interactions between mutations, so it is not clear how ecological processes (and stochasticity) affect how they accumulate.

Our results were robust to changes in a number of modeling assumptions, which indicates that our findings are likely to be quite general. Still, it is somewhat surprising that different forms of fitness costs (accelerating, decelerating, or linear) did not produce markedly different results, as different trade-off shapes are often associated with contrasting outcomes in studies of host and parasite evolution (Kisdi 2006; Best et al. 2010). This suggests that epistasis plays a dominant role in shaping host-parasite coevolution in our model.

The findings presented herein are consistent with recent empirical work showing that infectivity evolution tends to proceed faster in the presence of coevolving antagonists that exhibit gradual changes in phenotype (Poullain et al. 2008; Paterson et al. 2010; Schulte et al. 2010; Hall et al. 2011; Morran et al. 2011; Zhang and Buckling 2011). The host-parasite systems in these experiments appear to feature multiple reciprocal genetic adaptations, comparable to the symmetric (SYM) scenario in our model. Furthermore, some studies have focused specifically on the evolution of broad infectivity ranges, with parasites experiencing either coevolving or constant mixtures of host genotypes (Benmayor et al. 2009; Hall et al. 2011). The latter treatment is analogous to our asymmetric (ASYM) scenario, as parasites must adapt to a large phenotypic change in the host and do not have access to intermediate populations. Using the host bacterium *Pseudomonas fluorescens* and the lytic phage Φ2, Benmayor et al. (2009) manipulated the ratio of sensitive to resistant hosts and observed an apparent trade-off between mutation supply and selection for broader infectivity ranges, in much the same way as epistasis affected our ASYM scenario. Building on this work, Hall et al. (2011) demonstrated that phages evolved broader infectivity ranges when hosts were allowed to coevolve. This microbial system is known to exhibit strong positive epistasis between infectivity mutations (Scanlan et al. 2011), so these findings are in excellent agreement with the results of our stochastic simulations. In another recent study involving bacteria and viruses (Meyer et al. 2012), a lytic mutant of phage lambda required four mutations to infect resistant *Escherichia coli*. These mutations showed all-or-nothing (i.e., strongly positive) epistasis, but in contrast to studies on *P. fluorescens*, the acquisition of these mutations resulted from fitness benefits on a subpopulation of bacteria that had reverted to susceptibility. As such, broader infectivity ranges were again promoted by a gradual change in host phenotype, but this was facilitated by host polymorphism.

Several studies have explored the evolution of broader infectivity ranges, using either explicit genetics (as here; see also Sasaki 2000; Agrawal and Lively 2002, 2003; Fenton and Brockhurst 2007; Fenton et al. 2009, 2012) or by treating infectivity as a quantitative trait (e.g., Best et al. 2010). However, few theoretical studies have examined how different forms of epistasis influence parasite evolution. As an exception, Fenton and Brockhurst (2007) explored the role of accelerating, decelerating and linear fitness costs on coevolutionary dynamics in a GFG framework. Our study complements this work by focusing on the effects of epistasis on infectivity, while still allowing qualitatively different forms of fitness costs to exist. Similarly, studies focusing specifically on genetic factors that influence the evolution of infectivity ranges are rare. Poullain and Nuismer (2012) recently demonstrated that frameworks of infection genetics with overlapping ranges (e.g., gene-for-gene; Sasaki 2000) are better suited to allow adaptation to a novel host than if ranges are disjoint (e.g., matching alleles; Hamilton 1980), but the authors did not consider the effects of multilocus interactions, epistasis, or the rate of phenotypic change in the host on the evolution of infectivity ranges, as have been explored in the present study.

We have focused on how epistasis influences parasite evolution, as the emergence of broader infectivity ranges is of greater biological relevance to epidemiology and public health. Still, it is also important to understand how epistasis shapes host evolution, as this can elucidate patterns of selection among parasites (Supplementary Fig. S5). For example, hosts did not tend to become resistant in the stochastic SYM scenario when epistasis was negative, which explains why broader infectivity ranges did not evolve under these conditions. Host-parasite coevolution can lead to a variety of coevolutionary outcomes with respect to infectivity range, including competitive exclusion, stable polymorphism, and fluctuating selection (Sasaki 2000; Fenton and Brockhurst 2007). Indeed, our models produced fluctuations in range under certain conditions, but the primary purpose of our study has been to highlight conditions that promote or inhibit the accumulation of mutations that confer broader infectivity ranges, rather than whether they are evolutionarily stable. Hence, we chose to measure peak rather than final infectivity range, as the latter would have been heavily influenced by the choice of simulation length when fluctuations occurred. While evolutionary stability is often an important consideration, there are many circumstances where the initial emergence of a trait is more significant. For example, the chief concern in the context of emerging infectious diseases is whether a parasite will be able to cause an epidemic in a new population, rather than if it will survive over longer timescales. Compensatory mutations may also arise after a trait has initially spread, which could allow parasites to offset associated fitness costs. We hope to address questions surrounding the evolutionary stability of infectivity ranges under various genetic and ecological conditions in future work.

Understanding the effects of epistasis in real biological systems represents a significant challenge for empiricists and we are not aware of many nonmicrobial systems where the strength of interactions between infectivity mutations have been accurately measured (Hall and Ebert 2013). Finding a suitable host-parasite system to test the effects of weaker epistasis under different ecological conditions may prove to be difficult, but some of the predictions from our model should be relatively straightforward to verify experimentally with current approaches that utilize coevolving and noncoevolving communities.

## DATA ARCHIVING

The doi for our data is 10.5061/dryad.25kn0.

## Supporting Information

Disclaimer: Supplementary materials have been peer-reviewed but not copyedited.

Figure S1 Proportion of simulations where peak infectivity range (E; eq. 4) was greater than 0.9 (black), less than 0.1 (white), or between these values (gray) for *n* = 4.

Figure S2 Proportion of simulations where peak infectivity range (E; eq. 4) was greater than 0.9 (black), less than 0.1 (white), or between these values (gray) for density-dependent transmission (DD).

Figure S3 Proportion of simulations where peak infectivity range (E; eq. 4) was greater than 0.9 (black), less than 0.1 (white), or between these values (gray) for (a–d) linear (φ_H_=φ_P_=1) and (e–h) accelerating (φ_H_=φ_P_=2) fitness costs.

Figure S4 Proportion of simulations where peak infectivity range (E; eq. 4) was greater than 0.1 (black), less than 0.1 (white), or between these values (gray), for different combinations of fitness costs: (a–d) natural mortality (μ*_i_*) and transmission (β*_j_*) rates; (e–h) birth (*r*_*r**i*_) and disease-associated mortality (α*_j_*) rates; (i–l) natural (μ*_i_*) and disease-associated (α*_j_*) mortality rates.

Figure S5 Proportion of simulations where peak resistance range for hosts was greater than 0.9 (black), less than 0.1 (white) or between these values (grey). The measure used here for hosts is analagous to the one described for parasites in equation 4, with resistance substituted for infectivity.
