# Supplementary material for: EFFECTS OF EPISTASIS ON INFECTIVITY RANGE DURING HOST-PARASITE COEVOLUTION
Source: Evolution. 2014 Jul 25;68(10):2972–82. doi: 10.1111/evo.12479 (PMC4261995; doi:10.1111/evo.12479)
Supplement: Figure S2 — Proportion of simulations where peak infectivity range (E; eq. 4) was greater than 0.9 (black), less than 0.1 (white), or between these values (gray) for density-dependent transmission (DD). [file evo0068-2972-SD2.pdf]

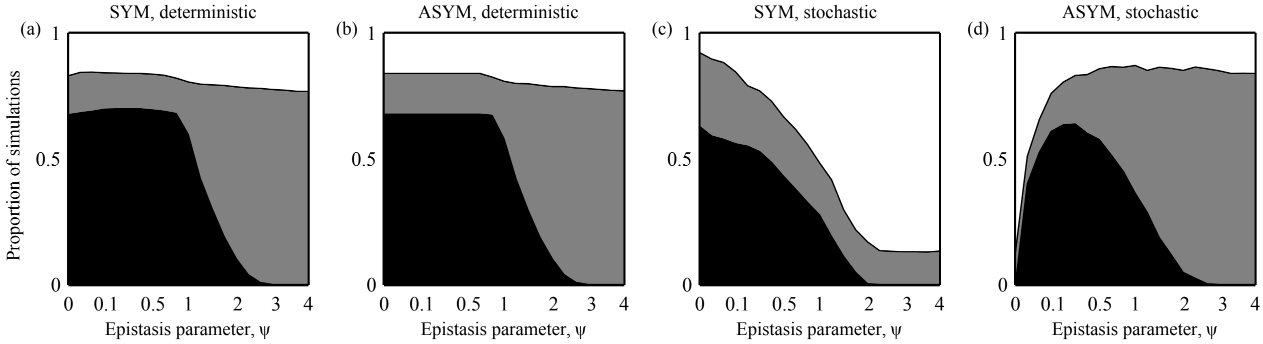

Figure S2 – Proportion of simulations where peak infectivity range (E; equation 4) was greater than 0.9 (black), less than 0.1 (white) or between these values (grey), for density-dependent transmission (DD). Plots (a-b) and (c-d) show results for deterministic and stochastic simulations, respectively. Plots (a, c) and (b, d) show results for gradual (SYM) and sudden (ASYM) changes in host phenotype, respectively. The parameter ψ controls the type and strength of epistasis between infectivity alleles, ranging from strong positive (ψ<<1), through weak positive (ψ<1), none (ψ=1) and finally, negative (ψ>1) epistasis. These results are broadly similar to those presented in the main text for frequency-dependent transmission (figures 3 and 4).
