# Supplementary material for: EFFECTS OF EPISTASIS ON INFECTIVITY RANGE DURING HOST-PARASITE COEVOLUTION
Source: Evolution. 2014 Jul 25;68(10):2972–82. doi: 10.1111/evo.12479 (PMC4261995; doi:10.1111/evo.12479)
Supplement: Figure S3 — Proportion of simulations where peak infectivity range (E; eq. 4) was greater than 0.9 (black), less than 0.1 (white), or between these values (gray) for (a–d) linear (φH=φP=1) and (e–h) accelerating (φH=φP=2) fitness costs. [file evo0068-2972-SD3.pdf]

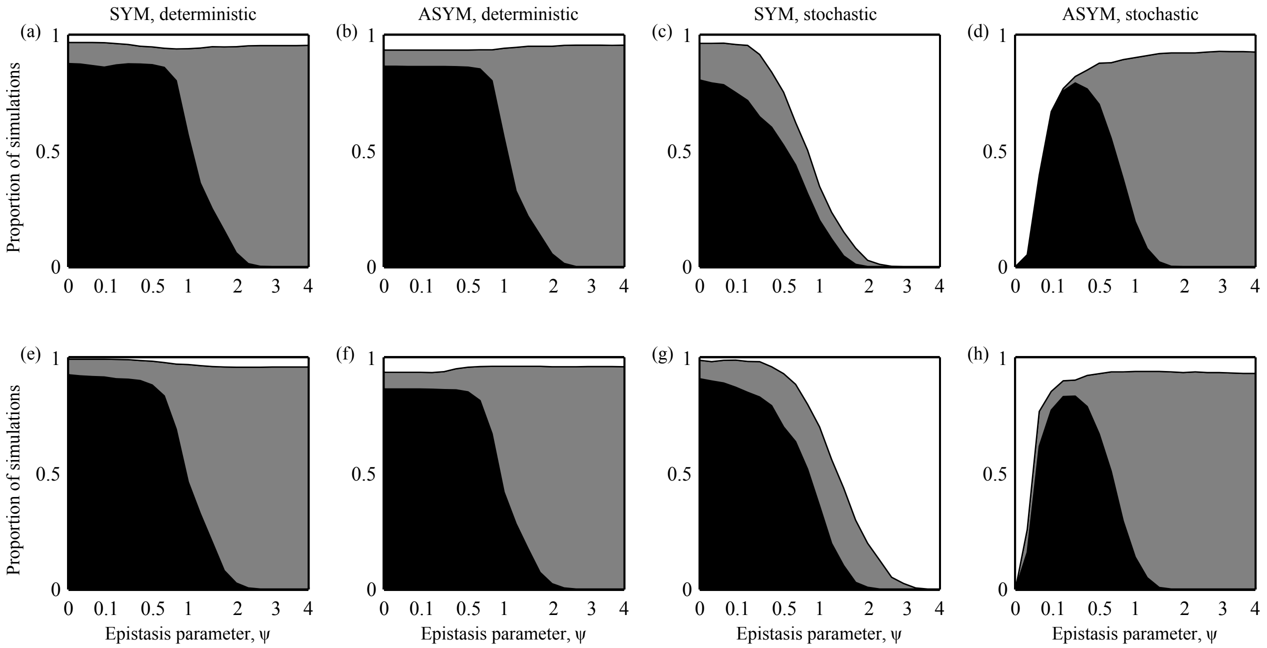

Figure S3 – Proportion of simulations where peak infectivity range (E; equation 4) was greater than 0.9 (black), less than 0.1 (white) or between these values (grey), for (a-d) linear (φ_H=φ_P=1) and (e-h) accelerating (φ_H=φ_P=2) fitness costs. The first and last two columns show results for deterministic and stochastic simulations, respectively. The first and third, and second and fourth columns show results for gradual (SYM) and sudden (ASYM) changes in host phenotype, respectively. The parameter ψ controls the type and strength of epistasis between infectivity alleles, ranging from strong positive (ψ<<1), through weak positive (ψ<1), none (ψ=1) and finally, negative (ψ>1) epistasis. These results are broadly similar to those presented in the main text for decelerating fitness costs (figures 3 and 4).
