# Supplementary material for: EFFECTS OF EPISTASIS ON INFECTIVITY RANGE DURING HOST-PARASITE COEVOLUTION
Source: Evolution. 2014 Jul 25;68(10):2972–82. doi: 10.1111/evo.12479 (PMC4261995; doi:10.1111/evo.12479)
Supplement: Figure S4 — Proportion of simulations where peak infectivity range (E; eq. 4) was greater than 0.1 (black), less than 0.1 (white), or between these values (gray), for different combinations of fitness costs: (a–d) natural mortality (μi) and transmission (βj) rates; (e–h) birth (rri) and disease-associated mortality (αj) rates; (i–l) natural (μi) and disease-associated (αj) mortality rates. [file evo0068-2972-SD4.pdf]

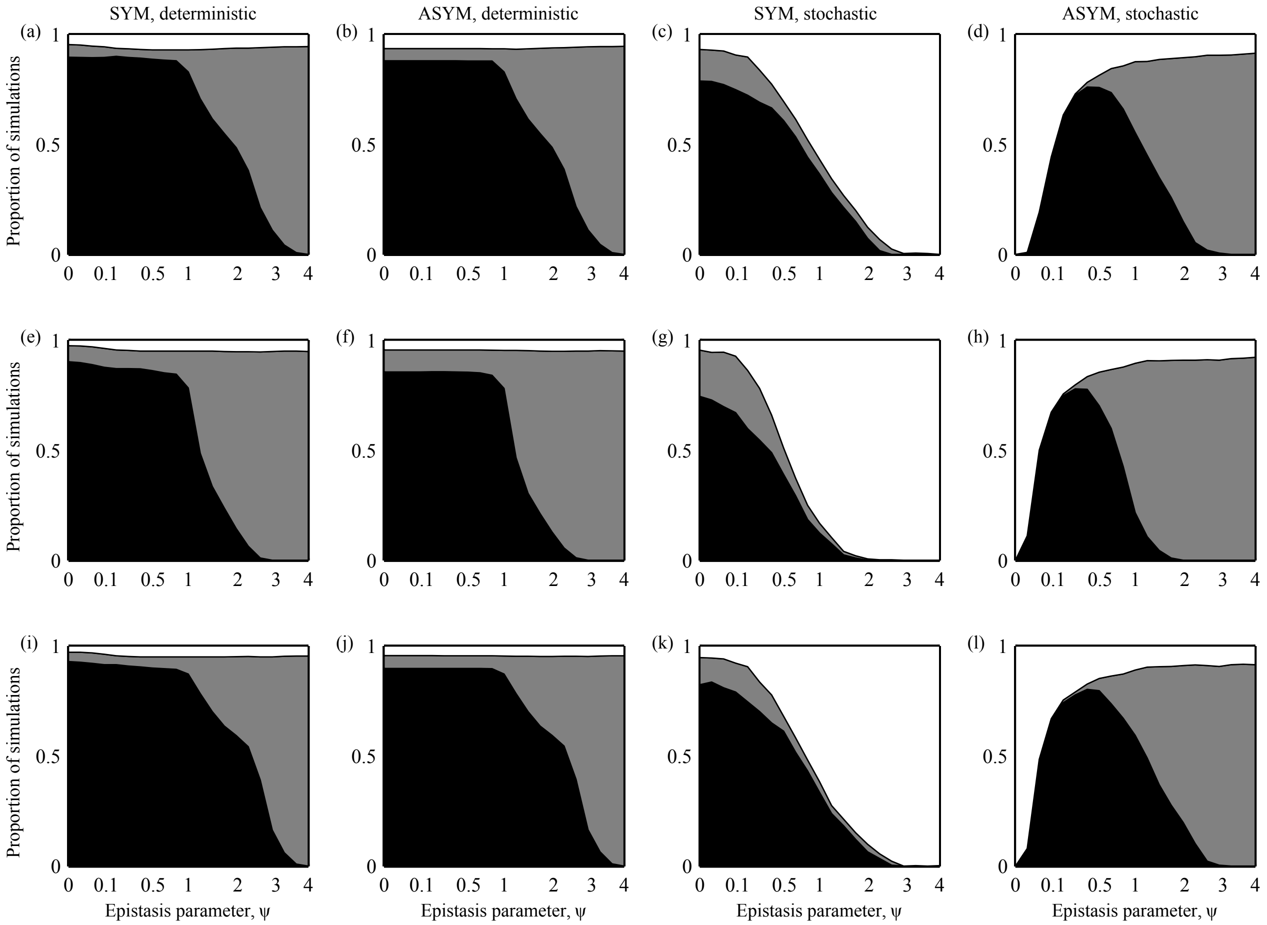

Figure S4 – Proportion of simulations where peak infectivity range (E; equation 4) was greater than 0.9 (black), less than 0.1 (white) or between these values (grey), for different combinations of fitness costs: (a-d) natural mortality (μ_i) and transmission (β_j) rates; (e-h) birth (r_i) and disease-associated mortality (α_j) rates; (i-l) natural (μ_i) and disease-associated (α_j) mortality rates. The first and last two columns show results for deterministic and stochastic simulations, respectively. The first and third, and second and fourth columns show results for gradual (SYM) and sudden (ASYM) changes in host phenotype, respectively. The parameter ψ controls the type and strength of epistasis between infectivity alleles, ranging from strong positive (ψ<<1), through weak positive (ψ<1), none (ψ=1) and finally, negative (ψ>1) epistasis. These results are broadly similar to those presented in the main text for fitness costs in the form of reduced birth (r_i) and transmission (β_j) rates (figures 3 and 4).
