# Supplementary material for: EFFECTS OF EPISTASIS ON INFECTIVITY RANGE DURING HOST-PARASITE COEVOLUTION
Source: Evolution. 2014 Jul 25;68(10):2972–82. doi: 10.1111/evo.12479 (PMC4261995; doi:10.1111/evo.12479)
Supplement: Figure S5 — Proportion of simulations where peak resistance range for hosts was greater than 0.9 (black), less than 0.1 (white) or between these values (grey). The measure used here for hosts is analagous to the one described for parasites in equation 4, with resistance substituted for infectivity. [file evo0068-2972-SD5.pdf]

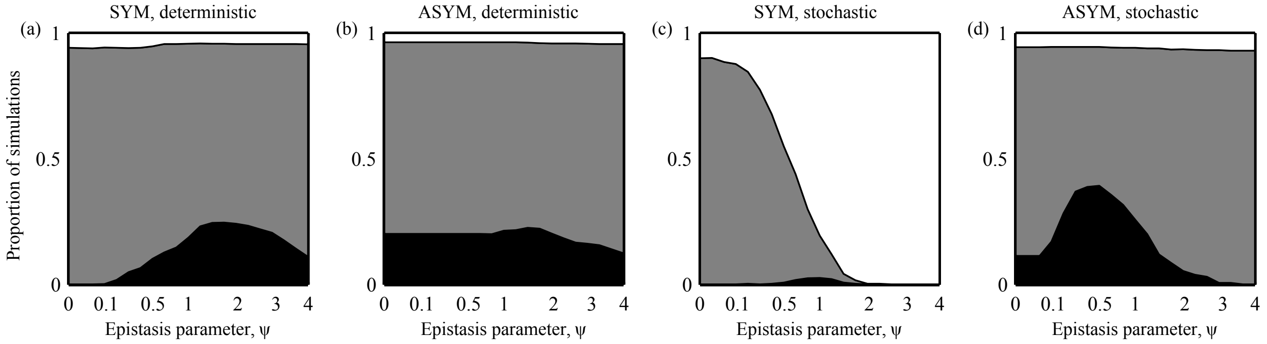

Figure S5 – Proportion of simulations where peak resistance range for hosts was greater than 0.9 (black), less than 0.1 (white) or between these values (grey). The measure used here for hosts is analagous to the one described for parasites in equation 4, with resistance substituted for infectivity. Plots (a-b) and (c-d) show results for deterministic and stochastic simulations, respectively. Plots (a, c) and (b, d) show results for gradual (SYM) and sudden (ASYM) changes in host phenotype, respectively. The parameter ψ controls the type and strength of epistasis between infectivity alleles, ranging from strong positive (ψ<<1), through weak positive (ψ<1), none (ψ=1) and finally, negative (ψ>1) epistasis.
